# The effects of drought on *Sphagnum* moss species and the implications for hydrology in peatlands

**DOI:** 10.1111/nph.70361

**Published:** 2025-07-07

**Authors:** Ben Keane, Danielle M. Alderson, Gareth D. Clay, Martin G. Evans, Chris D. Field, Adam Johnston, Juul Limpens, Colin P. R. McCarter, Nina Overtoom, Jonathan P. Ritson, Bjorn J. M. Robroek, Line Rochefort, Emma L. Shuttleworth, Yvet Telgenkamp, Merritt R. Turetsky, J. Michael Waddington

**Affiliations:** ^1^ School of Environment, Education and Development The University of Manchester Oxford Rd Manchester M13 9PL UK; ^2^ Department of Environment and Geography University of York York YO10 5DD UK; ^3^ Department of Geography Durham University Durham DH1 3LE UK; ^4^ Department of Natural Sciences Manchester Metropolitan University 6.21, Dalton Building, Chester Street Manchester M1 5GD UK; ^5^ JBA Consulting 1 Broughton Park, Old Lane North, Broughton Skipton BD23 3FD North Yorkshire UK; ^6^ Plant Ecology and Nature Conservation (PEN) Wageningen University & Research PO Box 47 6700AA Wageningen the Netherlands; ^7^ Department of Biology, Chemistry, and Geography Nipissing University 100 College Dr North Bay ON P1B 8L7 Canada; ^8^ Department of Ecology, Radboud Institute for Biological and Environmental Sciences Radboud University Heyendaalseweg 135 6681PR Nijmegen the Netherlands; ^9^ School of Biological Sciences University of Southampton SO17 1BJ Southampton UK; ^10^ Partnership Research in Ecosystem Restoration, Center for Northern Studies, Peatland Ecology Research Group Université Laval Quebec City QC G1V 0A6 Canada; ^11^ Department of Ecology and Evolutionary Biology, Renewable and Sustainable Energy Institute University of Colorado Boulder Boulder CO 80309 USA; ^12^ School of Earth, Environment & Society McMaster University Hamilton ON L8S 4L8 Canada

**Keywords:** carbon, drought, fire, hydrology, methane, peatland, restoration, *Sphagnum*

## Abstract

Peatlands store more carbon (C) than any other terrestrial ecosystem and as a C sink they are vital to mitigating climate change. The keystone of many peatland ecosystems is *Sphagnum*, a bryophyte genus of *c*. 350 species found on every continent except Antarctica. With climate change, many peatlands face increasing frequency and severity of drought. How *Sphagnum* responds to and recovers from drought will be key to sustaining peatlands over the coming decades. Here, we synthesise the latest evidence for how interactions of drought with *Sphagnum* affect peatland functioning. We discuss how *Sphagnum* traits, from the cellular to the community, control its ecohydrology and what changes occur during drought. We detail the effects of drought on *Sphagnum* C cycling and biochemistry, including photosynthesis, growth, respiration and methane (CH_4_) fluxes. We also highlight drought resilience and tipping points for *Sphagnum* physiology and at the ecosystem level. The implications of *Sphagnum* drought responses for peatland hydrology, restoration and wildfires are also outlined. Finally, we identify knowledge gaps and propose some urgent questions which should be addressed in future research.

**Table:** 

	Contents	
	Summary	2003
I.	Introduction	2004
II.	Community traits and ecohydrology under drought	2007
III.	Sphagnum C cycling and biochemistry under drought	2010
IV.	Implications for peatland hydrology: runoff, resilience, restoration and wildfire feedbacks	2015
V.	Future work	2016
	Acknowledgements	2016
	References	2017

## I. Introduction

**Fig. 1:**
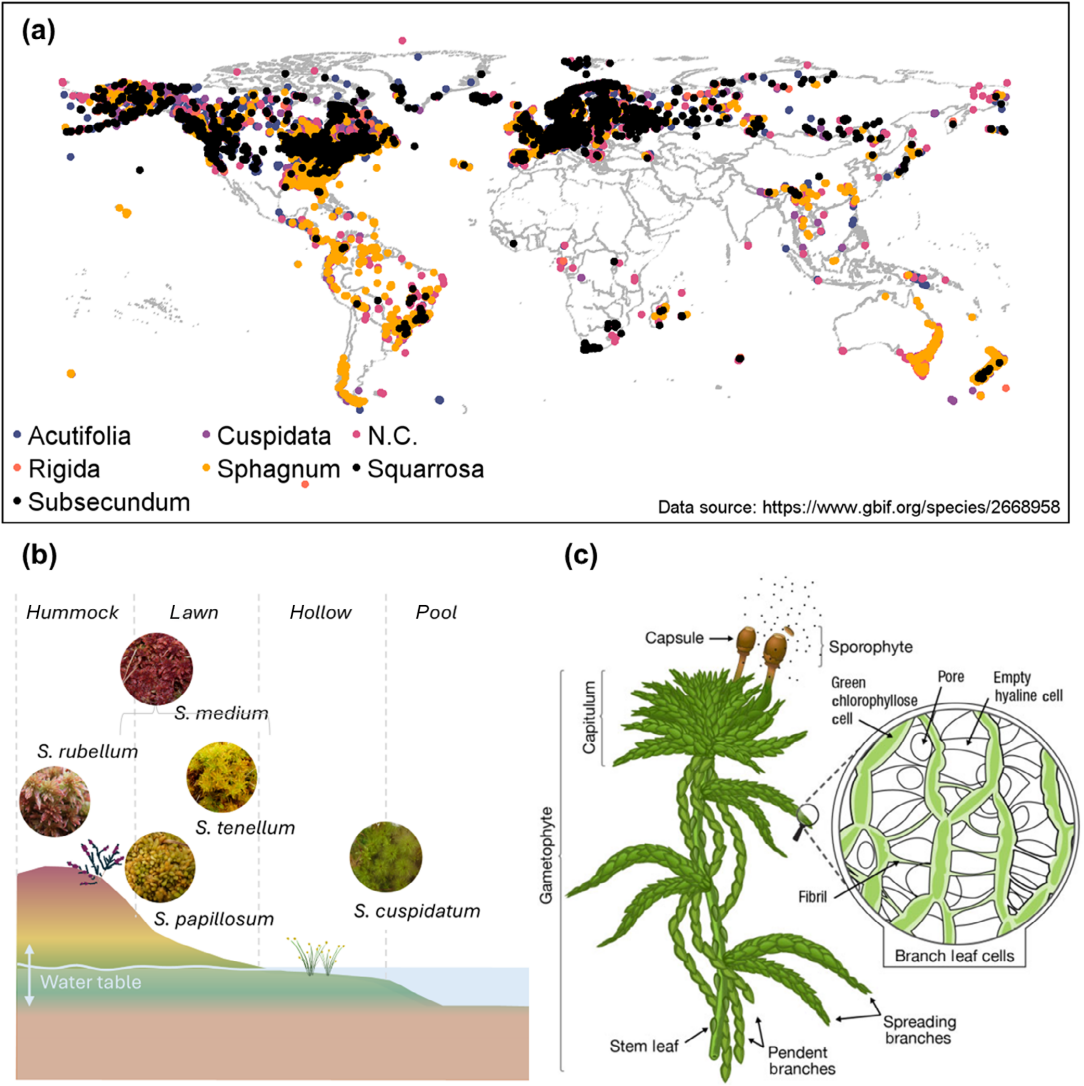
The global and local distribution of *Sphagnum* and a detailed desciption of its anatomy. (a) Global distribution of *Sphagnum* occurrences logged since 1950, excluding fossil and preserved samples. Data sourced from Global Biodiversity Information Facility. Colours respond to subgenus of *Sphagnum* (N.C. = not classified at the species level). (b) *Sphagnum* niches (with example species), hummock, lawn, hollow and pool, in relation to water table position. *Sphagnum* images by Sharon Pilkington and Paul Bowyer reproduced with permission from https://www.britishbryologicalsociety.org.uk. (c) Morphology of *Sphagnum*, sourced from (Weston *et al*., 2015).

Peatlands occur in wet environments where primary production exceeds microbial decomposition and combustion (Yu *et al*., 2010) and store between 450 000 and 650 000 megatons (Mt) of carbon (C) (UNEP, 2022). A more bold upper estimate suggested that this may be in excess of 1000 000 MtC (Nichols & Peteet, 2019) but has been called into question (Yu *et al*., 2021). A keystone group of species for the functioning of many of these ecosystems is *Sphagnum*, or peat moss, a distinct bryophyte genus consisting of over 350 species found across every continent except Antarctica (Fig. 1a). Although *Sphagna* predominantly occur at the high latitudes of the boreal zone of the Northern Hemisphere, they are also found in austral South America, Asia, Tasmania, New Zealand and Africa (Michaelis, 2019). It has been estimated that *Sphagnum* biomass exceeds that of any other land plant genus (Hayward & Clymo, 1982), making peatlands the largest C stock of the entire terrestrial biosphere (Scharlemann *et al*., 2014). *Sphagnum* performs several functions that engineer environmental conditions favourable to its proliferation:

1. Its nutrient use efficiency and interception of rainfall in ombrotrophic systems allow it to outcompete other plants in nutrient‐poor landscapes (Hayward & Clymo, 1982; Lamers *et al*., 2000).
2. Its high moisture content and capillary action maintain wet conditions, which reduce biomass decomposition and suppress the growth of vascular plants that can compete with *Sphagnum* for resources (Temmink *et al*., 2022).
3. *Sphagnum* creates acid conditions: This has been held widely to be due to directly releasing hydrogen (H^+^) and other cations into the environment (Clymo, 1963, 1987) although there is evidence that the release of organic acid from the breakdown of *Sphagnum* biomass is also important (Soudzilovskaia *et al*., 2010).
4. The high levels of polyphenolic compounds in its biomass mean that it is more recalcitrant, decomposing much slower than vascular plant material, leading to its accumulation as peat, which creates soil chemistry that suppresses vascular plant growth (Hájek *et al*., 2011).
5. It supresses microbial activity through release of specialised metabolites (Mellegård *et al*., 2009; Fudyma *et al*., 2019; Hamard *et al*., 2019).

This combination of traits results in *Sphagnum* being one of the most dominant plant functional types in many northern peatlands (Gunnarsson, 2005), making up much of the peat itself in boreal peatlands, upland blanket bogs and lowland raised bogs where they contribute a large percentage of the C store (Van Breeman, 1995).

**Table 1:** Details of *Sphagnum* drought experiments included in Figs 2 and 4.

References	Number	Year	Location	Method of water deprivation	No. of spp.	Drought duration (d)	Recovery duration (d)
Skre & Oechel (1981)	1	1981	Fairbanks Alaska	Desiccation	2	0.46	16.00
Wallen *et al*. (1988)	2	1988	South Sweden	WTD manipulation	3	1	
Hajek & Beckett (2008)	3	2008	Czech Republic	Desiccation	5	1.04	4.00
Hájek & Vicherová (2014)	4	2014	Finland, Slovakia	Desiccation	13	1.5	4.00
Murray *et al*. (1989)	5	1989	Alaska, USA	Desiccation	2	2	0.63
Rydin (1985)	6	1985	Eastern Sweden	WTD manipulation	4	3	
Vogelmann & Moss (1993)	7	1993	Maine and New Hampshire, USA	Desiccation	4	3	
Gerdol & Vicentini (2011)	8	2011	Italian Alps	Warming	2	4	6.00
Winnicka & Melosik (2019)	9	2019	Poland	Desiccation	1	4	7.00
Winnicka *et al*. (2020)	10	2020	Estonia	Desiccation	1	4	30.00
Winnicka *et al*. (2018)	11	2018	Poland	Desiccation	1	4	7.00
McNeil & Waddington (2003)	12	2003	Quebec, Canada	Desiccation	1	7	21.00
Salko *et al*. (2023))	13	2023	Southern Sweden	Desiccation	9	7	
Wagner & Titus (1984)	14	1984	New York, USA	Desiccation	2	10	1.25
Gerdol *et al*. (1996)	15	1996	Italian Alps	Desiccation	3	11	7.00
Schipperges & Rydin (1998)	16	1998	Northwest Sweden	Desiccation	4	12	0.02
Nibau *et al*. (2022)	17	2022	Wales	Desiccation	4	13	1.00
Jablonska *et al*. (2023)	18	2023	Poland	Desiccation	2	14	
Jassey & Signarbieux (2019)	19	2019	Jura Mountains, France	Natural drought	2	14	0.04
Moore *et al*. (2021)	20	2021	Ontario, Canada	Desiccation	2	14	
Sagot & Rochefort, 1996	21	1996	Canada	Desiccation	3	14	
Harris *et al*. (2005)	22	2005	Wales	Desiccation	2	15	6.00
Bengtsson *et al*. (2020)	23	2020	Southern Sweden	WTD manipulation	13	20	
Keane *et al*. (2021)	24	2021	Southwest Sweden	Natural drought	1	21	182.00
Verhoeven *et al*. (2021)	25	2021	Minnesota, USA	Desiccation	1	21	1.00
van de Koot *et al*. (2024)	26	2024	Wales	Desiccation	4	21	7.00
Harris (2008))	27	2008	Wales	Desiccation	5	23	
Robroek *et al*. (2009a)	28	2009	Ireland	WTD manipulation	3	24	
Goetz & Price (2016)	29	2016	Alberta, Canada	Rain exclusion	2	42	
Kokkonen *et al*. (2024)	30	2024	Southern Finland	WTD manipulation	7	43	21.00
Koronatova *et al*. (2022)	31	2022	Siberia	Natural drought	4	56	
Stuart *et al*. (2022))	32	2022	Peak District, UK	Desiccation	3	56	
Serk *et al*. (2021)	33	2021	Northern Sweden	WTD manipulation	2	70	
Bu *et al*. (2013)	34	2013	Northeast China	WTD manipulation	3	77	
Tucker *et al*. (2022)	35	2022	Michigan, USA	WTD manipulation	2	77	
Lees *et al*. (2020))	36	2020	Flow Country, UK	Desiccation	2	80	
Lees *et al*. (2019)	37	2019	Flow Country, UK	Desiccation	2	84	30.00
Jauhiainen *et al*. (1997)	38	1997	Finland	WTD manipulation	1	87	
Nijp *et al*. (2014))	39	2014	Southern Sweden	WTD manipulation	3	90	7.00
Strack *et al*. (2009)	40	2009	Quebec, Canada	WTD manipulation	1	90	
Newman *et al*. (2018)	41	2018	Northwest England	WTD manipulation	3	91	230.00
Estop‐Aragonés *et al*. (2016)	42	2016	Ireland, UK, Poland, Slovaki	WTD manipulation	1	100	230.00
Grosvernier *et al*. (1997)	43	1997	Switzerland	WTD manipulation	3	105	
Li *et al*. (1992))	44	1992	Michigan, USA	WTD manipulation	2	120	
Adkinson & Humphreys (2011)	45	2011	Ontario, Canada	Rain exclusion	2	150	
Bragazza (2008)	46	2008	Italian Alps	Natural drought	2	153	
Harris *et al*. (2005)	47	2006	Wales	Natural drought	1	182.5	
Robroek *et al*. (2007a)	48	2007	Ireland	WTD manipulation	4	182.5	
Sterk *et al*. (2023)	49	2023	Flow Country, UK	Natural drought	1	182.5	
Gaudig *et al*. (2020)	50	2020	Northwest Germany	WTD manipulation	4	280	
Kim *et al*. (2021)	51	2021	Canada	WTD manipulation	3	350	
Breeuwer *et al*. (2009)	52	2009	Southern Sweden	WTD manipulation	2	541.88	
Rastogi *et al*. (2019))	53	2019	Poland	Rain exclusion	2	730	
Rastogi *et al*. (2019)	54	2020	Poland	Rain exclusion	2	730	
Tilak *et al*. (2022)	55	2022	Ireland	Natural WTD	1	730	
Norby *et al*. (2019)	56	2019	Minnesota, USA	Warming	2	1095	
Robroek *et al*. (2009b)	57	2009	Ireland and Estonia	Natural WTD	3	1095	
Weltzin *et al*. (2001)	58	2001	Minnesota, USA	WTD manipulation	2	1095	
Karofeld *et al*. (2020)	59	2020	Estonia	Natural WTD	3	1460	
Köster *et al*. (2023)	60	2023	Finland	WTD manipulation	1	1460	16.00

WTD, water table depth.

Globally, 12% of peatlands have been described as degraded (Joosten, 2016), but the extent to which human activities have damaged peatlands may be considerably greater: For example, across Europe, this figure is estimated to be 25%, rising to 50% within the European Union (EU; Tanneberger *et al*., 2021c) to as much as 80% in the UK (Moxey & Moran, 2014) and 85% in Germany (Joosten *et al*., 2017). Other regions with severely deplete peatlands include southeast Asia (*c*. 45%; Joosten *et al*., 2017) and New Zealand (*c*. 90%; Peters & Clarkson, 2010). Concerted efforts to restore peatlands have recognised the need for healthy *Sphagnum* and often restoration efforts depend on the function of *Sphagnum* to be successful (Rochefort *et al*., 2003).

In addition to the damage from current and historic management, *Sphagnum* peatlands now face the ongoing threat of climate change. It is beyond doubt that human activities are responsible for global warming, driven by anthropogenic emissions of greenhouse gases (GHGs), such as carbon dioxide (CO_2_). Without active removal of GHGs from the atmosphere (Roy *et al*., 2018; Smith *et al*., 2019), warming will continue throughout the remainder of the twenty‐first century (Masson‐Delmotte *et al*., 2021). Of the techniques proposed for GHG removal, many countries are relying on nature‐based solutions, such as the C sink of peatlands to meet their Paris Agreement targets (Tanneberger *et al*., 2021a, 2021b). While historically terrestrial ecosystems sequester 112–169 Pg C yr^–1^ globally (Sha *et al*., 2022), in 2023 that figure was severely reduced (Ke *et al*., 2024), indicating that the terrestrial C sink, and particularly that of *Sphagnum* peatlands, is itself vulnerable to climate warming (Helbig *et al*., 2022; Virkkala *et al*., 2025) and drought specifically (Keane *et al*., 2021).

Critically, amongst the many harmful effects of climate change is warming, leading to increased drought (see tables 19.1 & 19.2 in Schneider *et al*., 2007). There are several types of drought, including meteorological, hydrological and agricultural. In this review, we focus primarily on meteorological drought, a reduction from average precipitation that may subsequently induce other forms of drought (Lloyd‐Hughes, 2014). However, we acknowledge that hydrological effects caused by drought (e.g. lowered water table) may also be caused although management, such as drainage, and the effects of drainage (amongst other managed anthropogenic disturbances), has been reviewed recently in some detail (Pacheco‐Cancino *et al*., 2024). Consequently, we also consider studies that mimic the hydrological effects of drought through manipulations, such as water table drawdown. Reduction in precipitation, alongside increased evapotranspiration in boreal (Vicente‐Serrano *et al*., 2022) and temperate areas, such as the UK (Parry *et al*., 2024), have caused drying across many northern peatlands over the last 300 yr (Swindles *et al*., 2019). While this may lead to increasing probability of drought in continental peatlands (Cook *et al*., 2020; Helbig *et al*., 2020), the picture is less clear for boreal and coastal peatlands, which may actually be getting wetter (Giese *et al*., 2025). Nevertheless, climate change is inducing drying in many *Sphagnum*‐dominated peatlands leading to uncertainty in their ability to remain net C sinks (Helbig *et al*., 2020). Thus, of all the terrestrial biomes, peatlands may be the most important yet vulnerable terrestrial C sink (Strack *et al*., 2022; Temmink *et al*., 2022).

How peatlands respond to drought stress will likely have implications for future climate. While at present, the C sink of peatlands acts as a global cooling mechanism. Should this sink shift to a source as some predict (Lund *et al*., 2012; Mueller & Joos, 2021), it may create a positive feedback loop leading to further warming (Salimi *et al*., 2021). Elsewhere, some peatlands in northern regions are becoming wetter, for example with increased subsidence and flooding triggered by permafrost thaw, with consequences for vegetation and methane emissions (Giese *et al*., 2025). There is still significant uncertainty to how peatlands will respond to future climate and hydrological variability (Ritson *et al*., 2025), with some model simulations predicting that they will remain C sinks and continue climate mitigation (Qiu *et al*., 2022). Additionally, peatland C stocks are still not accounted for in many earth system models (Loisel *et al*., 2021), which are a key tool for directing climate change mitigation. It is therefore vital to understand how these key ecosystems are affected by climate‐induced stresses, and perhaps the most important species–response combination to sustaining functioning peatlands is the response of *Sphagnum* to changes in hydrology.

Over the course of this review, we will describe key studies of drought effects in *Sphagnum*. The length of drought studies on *Sphagna* varies greatly, from a few hours (Skre & Oechel, 1981; Hajek & Beckett, 2008) to several years (Weltzin *et al*., 2001; Rastogi *et al*., 2019; Köster *et al*., 2023). Study length is related to the method of inducing drought: Desiccation studies, which tend to be performed on isolated plant material in microcosms, dominate the shorter experiments (e.g. Skre & Oechel, 1981; Vogelmann & Moss, 1993; Winnicka & Melosik, 2019), although they may run for several months (Lees *et al*., 2020); mesocosm studies, which most commonly manipulate WTD, usually endure several months (Robroek *et al*., 2007a), although they may be studied for several years (Weltzin *et al*., 2001); field studies (Köster *et al*., 2023), or *in situ* large plot manipulations (Breeuwer *et al*., 2009; Norby *et al*., 2019), may cover several growing seasons which may include several discrete drought periods. Studies which make use of natural drought occurrence often compare between years (Jassey & Signarbieux, 2019; Keane *et al*., 2021) or between geographic locations (Bengtsson *et al*., 2021).

**Fig. 2:**
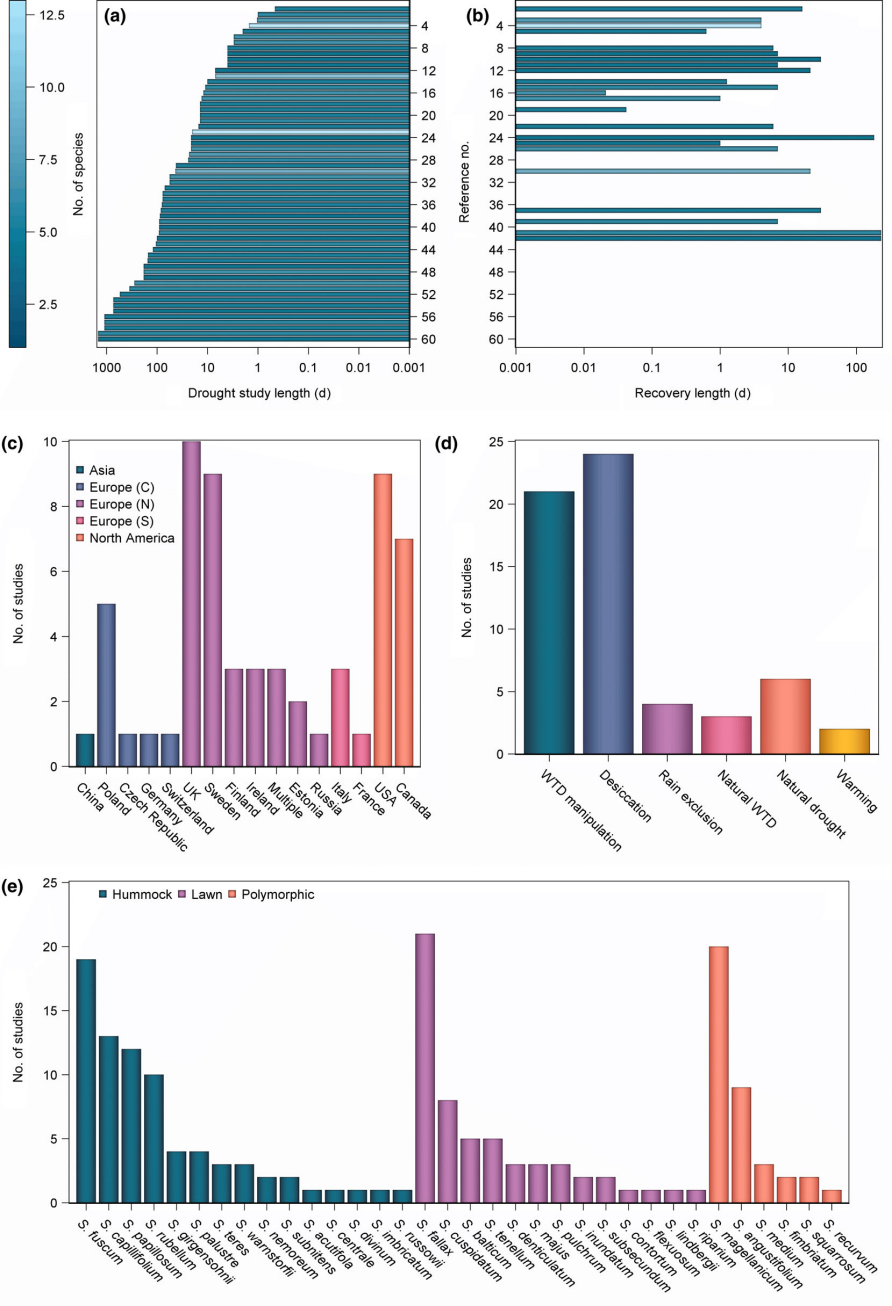
Summary of 60 (see Table 1) experimental drought studies retrieved from a Web of Science search for: *Sphagnum* AND (drought OR desiccation). Panels a and b show the length of the drought and recovery periods in each study and the number of *Sphagnum* species included. Panel c illustrates the location of the studies by country and geographic region. Panel d describes the type of experimental manipulation used to study the effects of drought. Panel e shows the number of times each species is represented in these studies and the ecotype each species presents.

The concept of recovery may be thought of as whether the ecosystem, community or species ‘bounces back’ following drought, and requires studying a period during which conditions return to predrought levels through, for example, rewetting by raising the water table or restoring rates of precipitation. Van Ruijven & Berendse (2010) define three related metrics to assess the impact of drought: resistance (change of a response variable from predrought to drought), recovery (change between drought period and postdrought) and resilience (change between predrought and postdrought), and these definitions have been used in subsequent drought studies (e.g. Kuiper *et al*., 2014; Kokkonen *et al*., 2024; Robroek *et al*., 2024). However, other studies simply discuss recovery as the comparison of postdrought to predrought (Nijp *et al*., 2014; Lees *et al*., 2019). Recovery is studied less often and for shorter periods than drought itself (Fig. 2). It may be studied at the level of a single response variable (e.g. photosynthesis, growth and moisture content), a selection of variables or the peatland ecosystem function as a whole. In reviewing the literature, we will discuss how these studies have assessed *Sphagnum* responses to drought and discuss the implications for restoration efforts, future climate and identify research priorities.

A note: *Sphagnum* research has been conducted for many decades, and the nomenclature surrounding some species has changed over time: For example, *S. medium* and *S. divinum* are two related species previously classified together as *S. magellanicum* (Hassel *et al*., 2018). For the purpose of this review, *Sphagnum* species are reported as they are described in the literature cited.

## II. Community traits and ecohydrology under drought

As nonvascular plants, *Sphagna* have no root system or stomata by which to self‐regulate their internal moisture. Instead, they have developed several unique adaptations which aid water retention and conveyance (Fig. 1c). At the cellular level, there is differentiation between photosynthetically active cells with chloroplasts and dead empty hyaline cells (Michaelis, 2019). Cells are supported by fibrils (Shaw *et al*., 2003), and pores in the hyaline cells enable water conductivity along the stems of *Sphagnum* (McCarter & Price, 2014). The majority of *Sphagnum* biomass is found in the leaves, which consist of a single layer of cells that alternates between large hyaline cells and smaller chlorophyllose cells (Clymo, 1970). Branches differentiate between hanging (pendent) and spreading, with the latter being particularly good at intercepting and retaining precipitation, and transferring water laterally between individual plants (Rice, 2012). Pendent branches, on the contrary, facilitate the capillary action of water upwards from the water table (Clymo, 1973; van de Koot *et al*., 2024). Collectively, this architecture creates a network of overlapping leaves, branches and pore spaces, trapping water extracellularly and internally within hyaline cells, so that *Sphagnum* moisture may be in excess of 20 times its dry biomass (Hayward & Clymo, 1982; Lees *et al*., 2019).

The branching density of *Sphagnum* can vary between species and within species depending on factors, such as seasonality (Clymo & Hayward, 1982). The lateral and vertical water transfer processes within *Sphagnum* peatlands therefore depend on the specific morphological traits of a given species or species assemblage. Above a spatial threshold of 25 × 25 cm (Clymo & Hayward, 1982), *Sphagnum* generally grows in dense multi‐species communities, with the shoot density controlled by factors, such as capitula size and rate of new capitula formation, which may be affected by genetics or micro‐scale environmental factors, such as distance from the water table (Elumeeva *et al*., 2011) or etiolation following flooding events (Rochefort *et al*., 2002). *Sphagnum* adapted to grow further from the water table, such as *S. fuscum*, form tight hummocks with high shoot density, while in hollows and pools closer to and below the water table, *Sphagnum* forms lawns where shoot density may be much lower as there is an abundant supply of water from the water table (McCarter *et al*., 2024). This leads to a characteristic hummock‐hollow microtopography seen in many peatlands (Fig. 1b), with the specific niche of individual species varying, for example along a chemical gradient from mineral rich fens and ombrotrophic bogs, between low and high pH, or a hydrological gradient between microtopographical features in the landscape (Weston *et al*., 2018). Coarsely, the genus *Sphagnum* can be divided into four subgenera, formally referred to as sections, which associate with differing microhabitat preferences. Typically, the subgenera *Cuspidata* and *Subsecunda* produce lawns or carpets and occupy hollows, whereas the subgenera *Sphagnum* and *Acutifolia* form hummocks. However, not all species within these sections conform to those niches, and others possess the plasticity to exhibit either ecotype and may be referred to as polymorphic (Piatkowski & Shaw, 2019).

During periods of drought, the water table in peatlands drops, increasing water scarcity. A common hypothesis is that hummock forming *Sphagnum* species are adapted to conditions further from the water table and therefore will be more resilient to periods of drought (to be described later in the section on drought mitigation). Hummock species (or more generally species that grow in a tighter community structure) will transport water more effectively at lower moisture contents from the water table or from extracellular storage, whereas lawn‐hollow species or communities have a more severe decline in the ability to supply water to the capitula during periods of little to no precipitation (Robroek *et al*., 2009a;McCarter & Price, 2014; McCarter *et al*., 2024). The leaf shape of *Sphagnum* species is important for water retention (Bengtsson *et al*., 2020), and it has also been demonstrated that *Sphagnum* adapts to dry conditions by increasing their internal storage capacity (number of hyaline pores) during drought (Li *et al*., 1992; Bu *et al*., 2013). The density of *Sphagnum* shoots may be key to drought response. For example, in *Sphagnum* hummocks (*S. fuscum*) shoot density may be > 300% greater than that of a *Sphagnum* lawn (*S. riparium*), enabling hummocks to access more water and retain it for four times as long under drought conditions (Elumeeva *et al*., 2011). The shoot density induces a difference in pore size distributions, where higher shoot densities, like *S. fuscum*, will have fewer macropores that readily drain with a receding water table than lower shoot densities like *S. magellanicum* (McCarter & Price, 2014; McCarter *et al*., 2024). The higher shoot density not only retains more water under drought, but has higher hydrological connectivity with the water table that facilitates better water supply to the capitula (McCarter & Price, 2014). It has been suggested that hummock *Sphagna*'s ability to transmit water creates the wet conditions that facilitate lawn‐hollow species’ survival (Wagner & Titus, 1984; Rydin, 1985, 1993), although experimental evidence indicates that this only occurs when precipitation remains providing moisture (Robroek *et al*., 2007b). In this light, further experiments have shown that during drought this ability instead enables hummock species to outcompete lawn‐hollow species (Bu *et al*., 2013). However, individual stems of *Sphagnum* species have also shown intrinsic abilities to tolerate prolonged period of desiccation or exposition to high temperature, with species like *S. fallax* having a greater power to regenerate postdesiccation than hummock species like *S. capillifolium* or *S. fuscum* (Sagot & Rochefort, 1996). The ability of some *Sphagnum* species to occupy both hummock and lawn ecotypes has been attributed to their ability to plastically change their morphology and consequentially soil hydraulic properties, depending on their specific environmental conditions (McCarter *et al*., 2024).

### 1. Mechanisms of drought mitigation

Plants use three broad strategies to protect themselves from drought: escape, through their life cycle; avoidance by excelling at water uptake and retention; and tolerance through physiology that tolerates desiccation (Zhang, 2007).

With regard to lifecycle, *Sphagnum* reproduces both asexually through fragmentation, which produces the majority of biomass in *Sphagnum* peatlands, and sexually, leading to the production of spores (Rydin *et al*., 2013). Production of spores may yield some protection against challenging conditions, allowing *Sphagnum* to escape periods of drought before germinating when conditions become more favourable (Fan *et al*., 2023). Despite dry‐wetting cycles diminishing the viability of *Sphagnum* spores, germination after > 10 yr has been identified in multiple *Sphagnum* species (Sundberg & Rydin, 2000).

Drought avoidance is the principal adaptation of *Sphagnum* (e.g. Bengtsson *et al*., 2020), which we know holds many times its dry weight in water. Hummock forming *Sphagnum* species are adapted to conditions further from the water table and therefore avoid drought by retaining moisture for longer (Keane *et al*., 2025). Drought avoidance in lawn‐hollow species relies on inhabiting wetter microhabitats close to the water table, which dry out less often (as mentioned in the previous section). Whether this translates into resilience of photosynthesis (and therefore C uptake) under drought conditions so that hummock species retain photosynthetic capability better under drought is less clear (Rydin, 1985; Robroek *et al*., 2024).

*Sphagna* have been thought to be relatively intolerant of drought, especially compared with other bryophyte genera which may survive years of desiccation (Stark *et al*., 2014). Inducible drought tolerance has been demonstrated in some species by ‘hardening’ during, for example frost (Campbell & Rydin, 2019) or water table lowering (Hájek & Vicherová, 2014). But while Hájek & Vicherová (2014) suggested inducibility was characteristic of lawn‐hollow species exclusively, a more recent study did not observe any difference in drought tolerance inducibility between hummock and hollow *Sphagna* (Kokkonen *et al*., 2024). Abscisic acid (ABA) is a plant signal, which stimulates stomatal closure in vascular plants to conserve water (Osmolovskaya *et al*., 2018), and recent work has shown that exposure ABA also increases drought tolerance in *Sphagnum* (Hájek & Vicherová, 2014). Furthermore, it has been shown that some *Sphagna* actively express the genes encoding ABA signalling (Nibau *et al*., 2022), and therefore, ABA itself may be an important indicator of drought in these mosses. ABA is not the only marker of potential drought tolerance: A ‘fingerprint’ of pigments and secondary metabolites associated with maintenance of photosynthesis has been identified in *Sphagnum* (Sytiuk *et al*., 2022, 2023), and may well be involved in drought resilience and therefore warrants further investigation. A number of genetic markers have also been identified, which are associated with drought stress in *Sphagnum* (Winnicka & Melosik, 2019), and recently, a hypothesis of ‘drought memory’ has been posited as an adaptation in *Sphagnum* (Kokkonen *et al*., 2024).

## III. *Sphagnum* C cycling and biochemistry under drought

### 1. Photosynthesis and growth

**Fig. 3:**
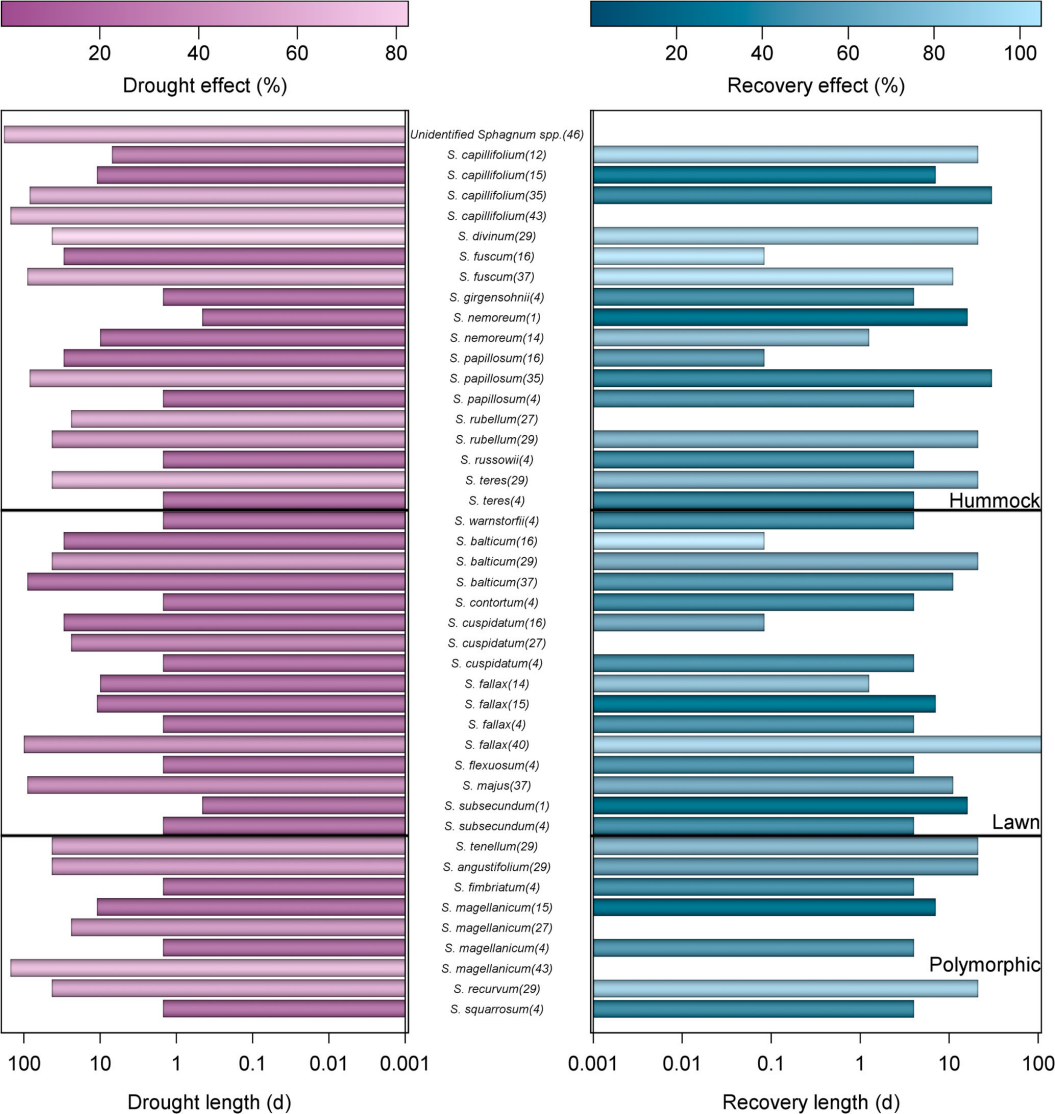
Decline and recovery of photosynthesis in drought studies of *Sphagnum* species as a function of study length as a proxy for drought intensity. Photosynthesis decline and recovery is expressed as a percentage of predrought CO_2_ fluxes to show effect of drought and extent of postdrought recovery. Note the log scale on the left‐hand panel. Numbers next to *Sphagnum* species name indicate the reference number from which the data were extracted (see Table 1).

After the withdrawal of external water through dropping the water table (e.g. Bengtsson *et al*., 2020), or in experimental desiccation studies (Gerdol *et al*., 1996; Stuart *et al*., 2022; van de Koot *et al*., 2024), *Sphagnum* begins to dry, reaching a moisture content asymptote any time between several hours (Schipperges & Rydin, 1998) and 2 wk (Lees *et al*., 2019) later. The rate at which *Sphagna* lose water varies with species, as does the optimum moisture level for CO_2_ uptake (Hayward & Clymo, 1982; McCarter & Price, 2014; Golubev *et al*., 2021; McCarter *et al*., 2024; Bengtsson *et al*., 2020). Both traits, alongside the severity of any drought period, will affect the magnitude of decline in CO_2_ exchange under drought conditions (Robroek *et al*., 2024). The optimum water content for *Sphagnum* photosynthesis tends to be in excess of 10 g g^−1^ of its own weight (Fig. 3), with hummock species, such as *S. palustre* and *S. fuscum* displaying maximum CO_2_ uptake *c*. 11 g g^−1^ (Bengtsson *et al*., 2020; Keane *et al*., 2025) compared with *c*. 14 g g^−1^ in lawn and polymorphic species, such as *S. balticum* and *S. magellanicum* (Gerdol *et al*., 1996; Schipperges & Rydin, 1998; Robroek *et al*., 2009a; Hájek, 2013; Nijp *et al*., 2014; Bengtsson *et al*., 2020; Keane *et al*., 2025). The relationship between moisture content and CO_2_ uptake tends to be asymmetrical, characterised by relatively stable rates of uptake above the optimum and a rapid decline below it, and thus, the optimum may also be viewed as a threshold (Gerdol *et al*., 1996; Schipperges & Rydin, 1998; Lees *et al*., 2020; Keane *et al*., 2025). In desiccation experiments, where *Sphagnum* is completely removed from a water source, rates of photosynthesis often decline severely to zero (Skre & Oechel, 1981; Schipperges & Rydin, 1998; Keane *et al*., 2025), whereas a lowered water table may reduce *Sphagnum* photosynthesis anywhere from a few percentage points to a complete shutdown (Strack *et al*., 2009; Robroek *et al*., 2009a; Nijp *et al*., 2014; Estop‐Aragonés *et al*., 2016; Kokkonen *et al*., 2024). Water table studies often express such thresholds in terms of water table depth (WTD) rather than moisture content of the *Sphagnum* itself, and the specific WTD of this threshold may differ between species. A WTD threshold of 55 cm was shown for the polymorphic *S. rubellum* (Strack *et al*., 2009), although a threshold WTD of *c*. 20 cm was shown for both the hummock species *S. fuscum* and the lawn species *S. magellanicum* (Strack & Price, 2009). Other studies express thresholds in terms of volumetric moisture content of the peat (e.g. Nijp *et al*., 2014), which was shown to be *c*. 0.48 m^3^ m^−3^ in the lawn species *S. balticum*. The scale of photosynthetic reduction under drought may be mitigated by precipitation: Even small amounts of rain, insufficient to completely raise the water table, may offset the reduction of CO_2_ uptake in these conditions (Robroek *et al*., 2009a; Adkinson & Humphreys, 2011; Nijp *et al*., 2014; Jassey & Signarbieux, 2019; Tucker *et al*., 2022). Similar reductions in photosynthesis have been seen in *Sphagnum* under natural drought conditions, such as that seen in northern Europe during the 2018 heatwave (Keane *et al*., 2021).

**Fig. 4:**
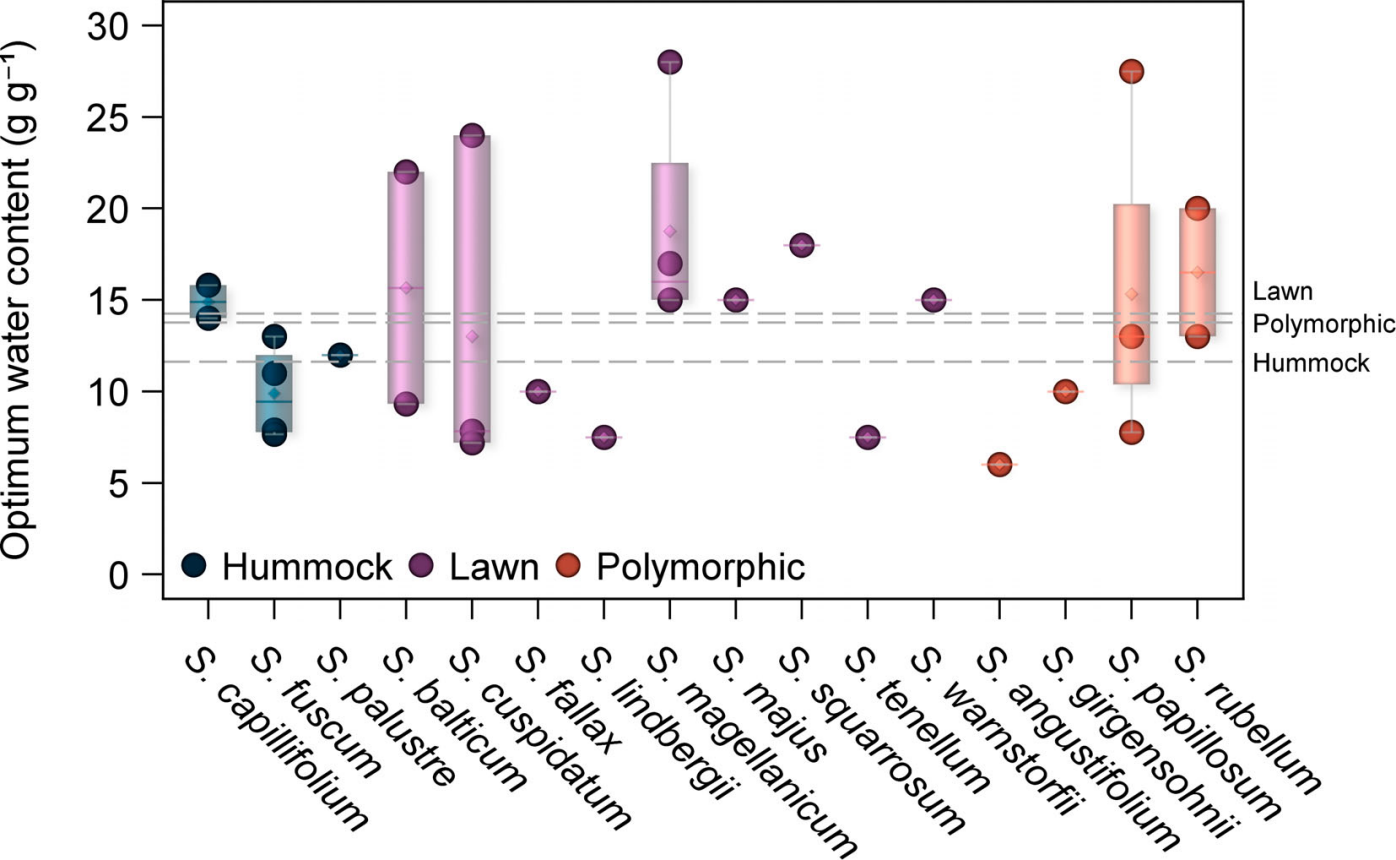
*Sphagnum* moisture content at which maximum CO_2_ uptake (optimum moisture content) has been measured experimentally in different *Sphagna*. Where multiple studies have reported data for a species, all data points are shown and the range (whiskers), median (diamond) and mean (horizontal line) are indicated by the box plots. Species are grouped by ecotype (hummock, lawn and polymorphic) and the overall mean for each ecotype is indicated by the dashed horizontal lines. Data from Gerdol *et al*. (1996); Schipperges & Rydin (1998); Lees *et al*. (2019); Bengtsson *et al*. (2020); Keane *et al*. (2025).

A well‐conserved characteristic across studies and species is rapid moisture uptake following rewetting, with predrought levels (or close to) reached usually within 1–2 d (Gerdol *et al*., 1996; Thompson & Waddington, 2008; van de Koot *et al*., 2024) even after repeated drying cycles (Schipperges & Rydin, 1998). Recovery of photosynthesis, however, shows large variation with length and severity of drought, both between and within species (Fig. 4). *S. capillifolium*, for example, demonstrated the ability to recover from a short drought of 1 wk after being rewetted for 20 d (McNeil & Waddington, 2003), but failed to recover from just 5 d of desiccation (Wagner & Titus, 1984) or a longer drought of 80 d, even after a 30‐d rewetting period (Lees *et al*., 2019). Another species which has been studied multiple times, *S. fallax*, recovered from 1.5‐d desiccation to *c*. 30% of its predrought photosynthesis with 4‐d recovery time (Hájek & Vicherová, 2014), but only recovered to 5% after 11 d desiccation and 1 wk's recovery period (Gerdol *et al*., 1996); over a much longer drought period (100 d), it recovered to 89% of its initial photosynthesis with 230‐d recovery (Estop‐Aragonés *et al*., 2016).

In studies comparing multiple species under the same conditions, differences are often reported between species. Nijp *et al*. (2014) found that one species, *S. fuscum*, made a full recovery after 11 d of drought, but two other *Sphagnum* species, *S. majus* and *S. balticum*, did not. Five *Sphagna*, including *S. magellanicum* and *S. cuspidatum*, failed to recover from a relatively short drought period (12 d; Schipperges & Rydin, 1998), but this may have been due to the short rewetting time of just 12 h; both species showed signs of recovering from a longer drought of 23 d, when given 16 d to recover (Robroek *et al*., 2009a). Another study of seven species presented a range of photosynthesis recovery, reporting a drop off of more than half (*S. angustifolium*) to nearly full recovery (*S. recurvum* and *S. divinum*; Kokkonen *et al*., 2024). An important point to consider when evaluating the responses presented in studies is that the desiccation responses of *Sphagnum* in isolation (i.e. studies on individual shoots) may be very different from the community response in a peatland environment (as mentioned in the earlier section on community ecohydrology; Sagot & Rochefort, 1996).

The effect of sequential desiccation and rewetting was shown to be detrimental to photosynthesis in a number of *Sphagnum* species (Schipperges & Rydin, 1998), declining by 50% by the fourth cycle in *S. balticum*, *S. cuspidatum* and *S. papillosum*; on the contrary, *S. fuscum* was robust to these cycles and maintained its predesiccation photosynthesis level throughout. This drop in photosynthesis has been shown to translate into growth, as Kim *et al*. (2021) showed a significant reduction in *Sphagnum* biomass under both fast and slow drought‐rewetting cycles compared with controls at constant moisture.

Although photosynthesis is linked closely to tissue moisture content, photosynthetic pigments, such as Chl and carotenoids, are responsible for the physiology of the process. Visually, the colour of vegetation can indicate pigment levels (e.g. greenness from Chl), and during drought a common response in *Sphagna* is ‘bleaching’, where the biomass becomes paler to the naked eye. A key example of this was during a natural drought of 2003, where such bleaching was observed for hummock species throughout the Italian Alps (Bragazza, 2008), and it is a phenomenon often reported in *Sphagnum* desiccation studies (Lees *et al*., 2019; Stuart *et al*., 2022). Changes in photosynthetic pigments can be quantified using nondestructive techniques, such as hyperspectral reflectance (Vogelmann & Moss, 1993; Harris, 2008) and Chl fluorescence (Hajek & Beckett, 2008), or destructively through analytical chemistry (Gerdol *et al*., 1996; Winnicka *et al*., 2020). Within a week of desiccation, Chl is known to drop by as much as 50% in *S. capillifolium*, although the *S*. *magellanicum* complex and *S. fallax* are more resilient, with *S. fallax* in particular maintaining predrying Chl levels for 11 d (Gerdol *et al*., 1996). However, in the same study, despite rewetting on the eleventh day, Chl levels continued to drop in *S. capillifolium* and *S*. *magellanicum*, and began to drop steeply in *S. fallax*, suggesting that irreversible damage could occur in all three species over this time (Gerdol *et al*., 1996). This points to a lasting drought legacy which can ultimately affect ecosystem functioning. Another species, *S. denticulatum*, displayed a complete shutdown of its photosynthetic apparatus within 3 d of desiccation (Winnicka *et al*., 2020). Rather than drought length, the severity of drying is perhaps a more useful indicator to consider. Nijp *et al*. (2014) identified a volumetric moisture threshold in peat of 0.48 m^3^ m^−3^ below which photosynthetic apparatus declined quickly to zero in *S. balticum* growing there. Looking specifically at *Sphagnum* tissue, Hajek & Beckett (2008) demonstrated a similar response in *S. fuscum* between 0.5 and 1.0 g g^−1^. Hyperspectral reflectance data can detect changes in photosynthetic pigments within the first days of drought before it is visually apparent (Stuart *et al*., 2022) and may be used to calculate several indices closely related to photosynthesis, such as normalised vegetation difference index (NDVI), photochemical reflectance index (PRI) and structurally insensitive pigment index, all of which significantly decline in drought conditions across a variety of *Sphagnum* spp. (Lees *et al*., 2019). These indices are useful tools for remote sensing, which can rapidly ascertain the drought status of large areas of *Sphagnum* landscapes (Harris *et al*., 2005; Lees *et al*., 2020) and can also differentiate between individual *Sphagnum* species (Newman *et al*., 2018; Lees *et al*., 2019; Salko *et al*., 2023).

Several studies have investigated the ability of photosynthetic pigments to recover following drought. Chl fluorescence recovered 90–100% in four species on rewetting (Nibau *et al*., 2022); this followed up to 13 d of desiccation, and considerable variation was reported for one species, *S. fallax*, which reflects the variation reported elsewhere in this species' recovery of photosynthesis (as mentioned in the previous paragraph). Rapid desiccation (< 24 h in an exsiccator) of four *Sphagnum* species caused a large variation in the recovery of Chl reflectance (from < 10% to *c*. 85%) both between and within species (Hájek & Vicherová, 2014). Similarly, the vegetation indices that are calculated as a proxy for photosynthesis also vary in their recovery from drought, depending on species and drought length. After 1 wk of drought, NDVI recovered in *S. palustre* but not in *S. squarrosum*, even after a 10‐wk recovery period (Keane *et al*., 2025). Neither *S. capillifolium* nor *S. papillosum* recovered to predrought NDVI values after rewetting for over a month following 80 d of drought (Lees *et al*., 2019). This indicates that, to varying degrees, drought damages the photosynthetic apparatus of *Sphagnum* species, the extent of which will constrain its ability to recover (Bragazza, 2008).

A trait closely linked to photosynthesis, and a key part of C accumulation in peatlands, is plant growth. Growth rates in a variety of *Sphagna* have been shown to decline with the severity of the drought (Li *et al*., 1992; Jauhiainen *et al*., 1997; Bu *et al*., 2013; Gaudig *et al*., 2020), although in one study, *S. fuscum* maintained its growth rate (Grosvernier *et al*., 1997). During times of drought, *Sphagnum* switches from using the C fixed by photosynthesis for growth, to storing it as nonstructural carbohydrates (NSCs) to maintain osmotic pressure and cell turgor (Chen *et al*., 2024). Furthermore, as well as drought inhibiting *Sphagnum* growth, it accelerates decomposition of *Sphagnum* necromass, through increased microbial phenol oxidase activity (Fenner & Freeman, 2011), leading to additional C losses (Kim *et al*., 2021). Overwhelmingly, the evidence suggests that the outlook for the C balance of *Sphagnum* dominated peatlands under drought is negative. There is, however, some suggestions that *Sphagnum* C losses may be counterbalanced by increased microbial C fixation under the warming, which tends to accompany drought (Le Geay *et al*., 2024).

**Fig. 5:**
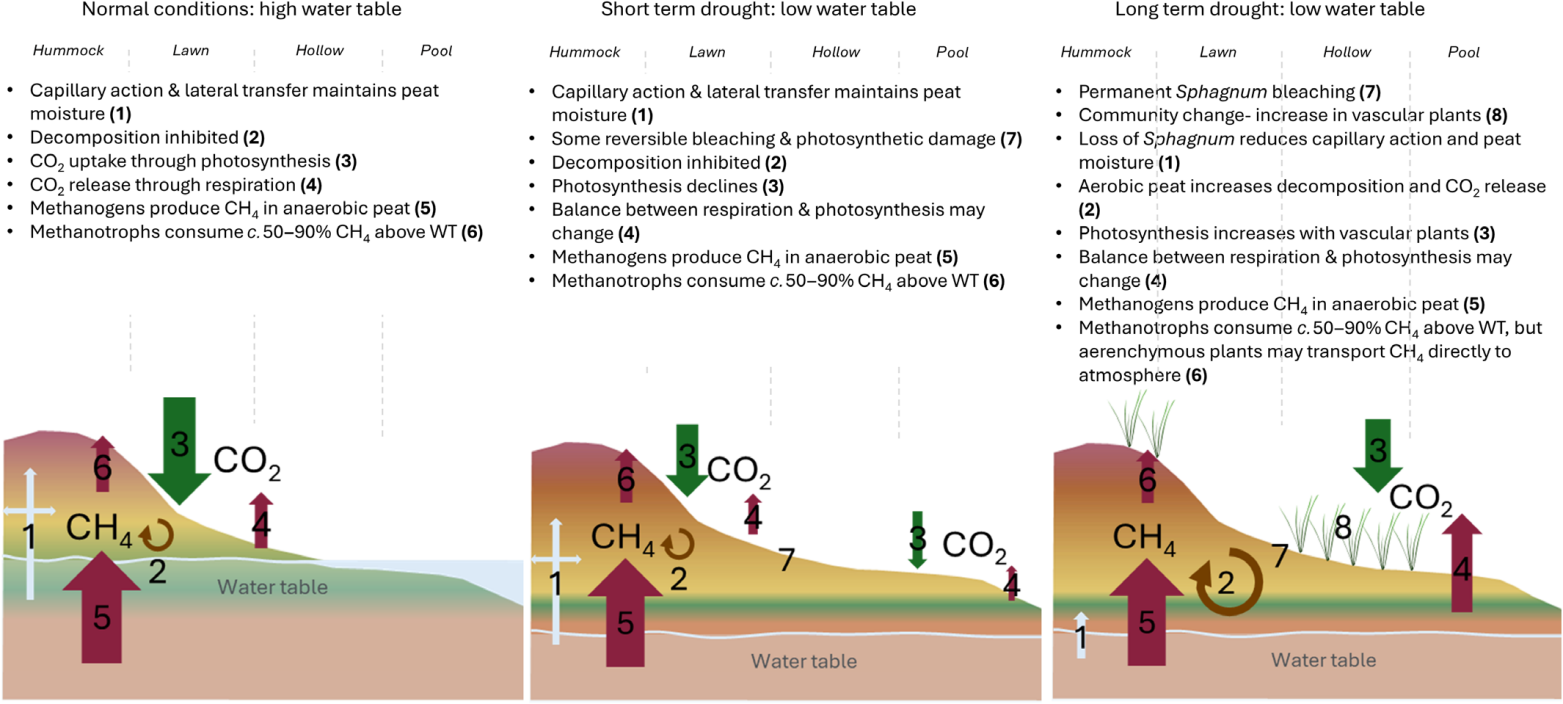
Conceptual overview of short‐ and long‐term effects of drought on *Sphagnum* peatland C cycling. The size of arrows represents nonscaled relative indicative magnitudes of fluxes.

Bragazza's (2008) study identified die‐off in *Sphagnum* hummocks through visual bleaching, calculating a precipitation to temperature ratio below which *Sphagnum* did not recover, even after several years. Following this, experimental work has identified thresholds on various aspects of *Sphagnum* physiology. A rapid drop in Chl reflectance was reported in *S. fallax* and *S. palustre*, with thresholds between 1% and 10% volumetric water content (Moore *et al*., 2021). A sharp drop in Chl reflectance was also seen in some (hollow) species below a threshold of *c*. 10 g g^−1^ moisture content (Bengtsson *et al*., 2020). These thresholds occur around the same moisture content as the optimum for photosynthesis, which has been reported to drop off rapidly below a moisture threshold in an asymmetric relationship in several *Sphagnum* species (Murray *et al*., 1989; Schipperges & Rydin, 1998; Thompson & Waddington, 2008). Below these optima, permanent damage to photosynthetic pigments has been reported; furthermore, this has been shown to be species‐specific, with the threshold lower in *S. palustre* than *S. squarrosum* (Keane *et al*., 2025), suggesting a stronger drought tolerance in the former. The permanent damage is likely due to the build‐up of reactive oxygen species (ROS) under the oxidative stress induced during drought (Halliwell & Gutteridge, 2015). Photosynthesis is not the only process affected by moisture thresholds. When the water table dropped below *c*. 20 cm, *Sphagnum* was replaced by graminoids, the microbial community altered, thus altering extracellular enzyme activity leading to a linear increase in ecosystem respiration (Jassey *et al*., 2018). These thresholds all have implications for the persistence of *Sphagnum* peatlands. The shift from *Sphagnum* to vascular plants under persistent high temperatures and reduced precipitation leads to reduced C accumulation and long‐term storage in these systems (Fig. 5; Bragazza *et al*., 2016). Several studies using palaeoecological techniques have been able to describe how the plant communities have changed over several millennia and have shown that hydrological changes have led to considerable changes (orders of magnitude) in the amount of *Sphagnum* cover within these landscapes (Lamentowicz *et al*., 2016; Swindles *et al*., 2019). A further study identified a tipping point of *c*. 11 cm WTD, below which vascular plant cover in proliferated in *Sphagnum* peatlands, although the authors suggest that due to the wide variety within the *Sphagnum* genus, the C sink functionality may be sustained past this threshold (Lamentowicz *et al*., 2019).

### 2. Respiration

While it is clear that drought significantly reduces photosynthesis, it is the balance between photosynthesis and respiration that governs the net balance of CO_2_. It is, therefore, important to understand how *Sphagnum* respiration is affected by drought. In both *S. subsecundum* (Skre & Oechel, 1981) and *S. fallax* (Skre & Oechel, 1981; Estop‐Aragonés *et al*., 2016), photosynthesis completely shut down after 16–100 d of no external precipitation, and concurrently, respiration increased leading to a net release of CO_2_ although the latter study measured ecosystem respiration and not from the *Sphagnum* alone. The opposite effect has been reported from several other *Sphagnum* species under drought conditions, where respiration has declined linearly with photosynthesis (Lees *et al*., 2019; Kokkonen *et al*., 2024) or even stopped completely (Keane *et al*., 2025).

Recovery of *Sphagnum* respiration is not consistent between studies. In some cases where respiration dropped during drought, it has been seen to recover to similar levels as predrought (Kokkonen *et al*., 2024). Elsewhere, respiration more than doubled during a drought period, only to fall back to predrought levels upon rewetting (Estop‐Aragonés *et al*., 2016). In other circumstances, particularly desiccation studies, rewetting of *Sphagnum* has led to a burst of respiration that far exceeds that of predrought levels (Robroek *et al*., 2009a; Lees *et al*., 2019; Keane *et al*., 2025). The postrewetting burst may be of microbial origin (metabolising contents of lysed *Sphagnum* cells; Gerdol *et al*., 1996) or autotrophic respiration resulting from *Sphagnum* diverting resources towards cell repair following drought‐induced damage (Robroek *et al*., 2009a).

### 3. Impacts on methane dynamics

Another important part of the peatland C cycle is methane (CH_4_) flux, with northern peatlands, including *Sphagnum* bogs emitting *c*. 11% of global emissions (Abdalla *et al*., 2016). Methane is produced through methanogenesis and consumed through methanotrophy, with the net flux the balance between the two processes. While aerenchymous peatland graminoids, such as *Carex* and *Eriophorum*, help transport CH_4_ from below the water table to the atmosphere (Keightley *et al*., 2023), *Sphagnum* has been shown to host methanotrophic microorganisms (Kip *et al*., 2010), which reduce the net emissions of CH_4_ from wetlands (Larmola *et al*., 2010) and may consume up to 90% of methane produced in *Sphagnum* peatlands (Kolb & Horn, 2012). Data are more scarce for CH_4_ fluxes than for CO_2_ exchange, but when *Sphagnum* has been studied in isolation it has been shown that CH_4_ uptake may increase under drought conditions by as much as 400% (Tucker *et al*., 2022). Where *Sphagnum* has been exposed to drought conditions in nonmonoculture mesocosms, the net CH_4_ flux also declines, but the effect is of reducing emissions rather than increasing uptake (Keane *et al*., 2021; Tucker *et al*., 2022). Since most studies do not partition the net flux into emission and uptake, it is difficult to conclude whether the effects observed in these studies are the result of an increase in methanotrophy, a reduction in methanogenesis or a combination of both.

Methane (CH_4_) fluxes have been shown to decline under drought, but this does not persist following rewetting. In a Swedish bog, CH_4_ emissions dropped from *Sphagnum* carpet during a natural drought, only to increase the year following the drought (Keane *et al*., 2021); at the same site, the microbial community was monitored over that period and, while not specific to the *Sphagnum*, it was shown that both the methanogens and methanotrophs were resilient to the drought (White *et al*., 2023). Peaks in CH_4_ emissions during postdrought rewetting have also been reported from other sites (Turetsky *et al*., 2014). It has also been shown that drought does not negatively affect the absolute abundance of bacteria associated with *Sphagnum*, although it does increase the abundance of fungi and therefore the fungal to bacterial ratio (Kim *et al*., 2021). This would suggest that the *Sphagnum* microbiome may be expected to retain its functionality upon rewetting, at least in the medium term. In the short term, the scant data available suggest that desiccation ‘switches off’ methanotrophy (Keane *et al*., 2025). Recalling that the postrewetting respiration burst seen from *Sphagnum* (as mentioned in the previous section) may be stimulated by the availability of low‐weight NSCs produced during drought, following *Sphagnum* cell damage during desiccation, there may be a competitive advantage for microbes to switch from metabolising CH_4_ until this alternative C source is exhausted.

### 4. Biogenic volatile organic compounds

In addition to GHGs, *Sphagna* also directly influence fluxes of biogenic volatile organic compounds (BVOCs; Janson & De Serves, 1998), a group of reactive hydrocarbons which are emitted by most terrestrial plants. The most common BVOCs associated with *Sphagna* are isoprene (Janson & De Serves, 1998; Männistö *et al*., 2023), monoterpenes and sesquiterpenes (Faubert *et al*., 2010). Although BVOC fluxes (μg C m^−2^ h^−1^) (BVOCs) are several orders of magnitude smaller than GHG fluxes (g C m^−2^ h^−1^ (CO_2_)), they are important due to their reactions in the atmosphere. BVOCs can exert a warming effect through competing for OH radicals in the atmosphere, enabling ozone formation in the troposphere (Atkinson, 2000) and also increasing the residence time of the GHG CH_4_ (Kaplan *et al*., 2006). Overall, however, BVOCs are thought to have a net climate cooling effect largely as a consequence of the secondary organic aerosols that they form (e.g. Hellén *et al*., 2020).

In a Swedish mire (68°20′N, 19°03′E), within species differences have been shown in BVOC flux, with *S. balticum* from wetter lawns emitting *c*. 30% more isoprene than *S. balticum* from dryer hummocks (Ekberg *et al*., 2011). The same study also demonstrated differences between species, with the hummock species *S. rubellum* emitting more BVOCs than the lawn species *S. magellanicum* (Ekberg *et al*., 2011). Where Ekberg *et al*. (2011) measured a maximum flux of > 100 μg C m^−2^ h^−1^, lower fluxes were recorded from a mixed *Sphagnum* lawn in an Alaskan tundra system at a similar latitude (68°38′N, 149°38′W), peaking at 38 μg C m^−2^ h^−1^, which constituted < 10% of the total ecosystem BVOC flux (Potosnak *et al*., 2013). *S*. *majus* was also shown to contribute < 10% of the total BVOC flux from a mire in central Finland (Tiiva *et al*., 2009).

BVOC flux has been positively related directly to photosynthesis (Ekberg *et al*., 2011) and therefore may be expected to decrease under drought conditions. This has been demonstrated, with total BVOC flux reduced by > 90% from both *S. balticum* and *S. recurvum* during a drought experiment (Männistö *et al*., 2024), although this study did not show any difference in the drought effect between *Sphagna* sourced from a minerotrophic fen or an ombrotrophic bog.

## IV. Implications for peatland hydrology: Runoff, resilience, restoration and wildfire feedbacks

Both *Sphagnum* peatlands' ability to slow‐release pore water during droughts and their ability to attenuate floods (natural flood management (NFM)) are two sides of the same coin. *Sphagnum* peatlands, therefore, are important for managing catchment hydrology naturally. While a proportion of NFM may be attributed to storage within the peat itself, particularly in peatlands with low to no topographic slope, the high water tables’ characteristic of intact *Sphagnum* peatlands mean that, rather than storing large amounts of storm precipitation, excess overland flow occurs quickly in these ecosystems (Evans *et al*., 1999). It is the surface roughness that *Sphagnum* generates that effectively reduces this overland flow in sloped peatlands (Holden *et al*., 2008), which increases lag time between storm events and peak discharge, and reduces that magnitude of peak discharge through a catchment, thus reducing the risk of flooding (Shuttleworth *et al*., 2019; Goudarzi *et al*., 2024). As such, how drought affects the growth and morphology of *Sphagnum* may have important implications for the flood alleviation provided by peatlands. Additionally, the large amount of water stored within *Sphagnum*, even when under drought conditions, results in a more consistent release of water during drought periods to downstream systems. As such, the overarching effect of *Sphagnum* cover is that downstream water supply can be more consistent, while also providing flood attenuation. A drop in growth and, if drought is severe enough, complete die‐off of some species (Bragazza, 2008) will reduce the capacity of *Sphagnum* peatlands to impact catchment‐scale hydrological processes, likely leading to more overland flow. This may become particularly problematic if weather patterns of increased drought and more intense rainfall events manifest over the coming decades. With prolonged drought conditions, it is likely that *Sphagnum* cover will shift towards vascular plant species, such as graminoids (Jassey *et al*., 2018; Buttler *et al*., 2023).

Wildfires are a major disturbance factor in northern peatlands (Turetsky *et al*., 2002), and burn frequency and severity (measured as depth of peat burned) can both increase significantly during drought conditions (Benscoter *et al*., 2011; Thompson *et al*., 2015; Wilkinson *et al*., 2019). Given that meteorological drought frequency and magnitude are expected to increase with climate change (Helbig *et al*., 2020), there are important direct and indirect hydrological implications for the interaction between drought, *Sphagnum* and wildfire (Turetsky *et al*., 2002). *Sphagnum* peatlands are generally resilient (Wieder *et al*., 2009) and in some cases resistant (Shetler *et al*., 2008; Lukenbach *et al*., 2015) to burning. The high moisture retention trait of *Sphagnum* limits the loss of C from peatlands by resisting ignition (Benscoter *et al*., 2011; Thompson *et al*., 2015; Wilkinson *et al*., 2019): The highest moisture content at which *Sphagnum* will ignite has been investigated experimentally, with one estimate to be 80% (and 71% at which it will sustain the ignition; Santana & Marrs, 2014); elsewhere an upper limit of 295% was identified (Benscoter *et al*., 2011), which is still well below the normal range, even in drought conditions. This might suggest that the fire risk of *Sphagnum* peatlands will not change under drought conditions. However, *Sphagnum* is often present in forests (e.g. Canadian spruce forests; Shetler *et al*., 2008) or in plant communities alongside shrubs and other readily flammable fuel sources; if fire does take hold during drought conditions, the sustained heat may create conditions in which *Sphagnum* will also ignite. Even under those circumstances, *Sphagnum* hummocks, such as those dominated by *S. fuscum*, appear to be more resilient to fire than other *Sphagnum* species, but the level of damage incurred increases in drier conditions (Andersen *et al*., 2024). Once a peatland surface burns, it can take decades for *Sphagnum* cover to re‐establish (Benscoter & Vitt, 2008; McDougall *et al*., 2023; Mayner *et al*., 2024). Near‐surface peat becomes increasingly hydrophobic with burn severity (Kettridge *et al*., 2014; Moore *et al*., 2017), inhibiting rewetting after fire and thus retarding postfire *Sphagnum* recovery and growth (Gage *et al*., 2024). That said, hydrophobic peat can act as a peatland water conservation system during drought through reduced evaporation (Kettridge & Waddington, 2014) although this feedback may break down with extreme burn severity (Kettridge *et al*., 2019). Nevertheless, if wildfire frequency and intensity increase with drought, so too will the prolonged damage to *Sphagnum* and the concomitant hydrological services associated with it.

Given the myriad of ecosystem services provided by ‘functioning’ peatlands, not least the C sequestration which many nations are relying upon to meet their climate commitments (Tanneberger *et al*., 2021b), there has been a drive to restore peatlands where they have been degraded. When restoring degraded peatlands, where *Sphagnum* has been lost through for example commercial peat extraction (Canada; González & Rochefort, 2014) or industrial pollution (UK; Lee *et al*., 1987), *Sphagnum* cover may remain < % and the landscape be infiltrated instead by vascular plants, such as birch without active *Sphagnum* reintroduction (Rochefort, 2000). In this respect, in many peatlands *Sphagnum* is the keystone for restoring sustainable peatland functioning. For successful restoration, it is vital we understand how reintroduced *Sphagnum* will respond to drought of different severities. A diverse selection of hummock and lawn species speed up the development of the C sink of restored *Sphagnum* peatlands (Robroek *et al*., 2009b). However, restoration of the Bois‐des‐Bel peatland in Canada introduced two species (*S. fuscum* and *S. rubellum*), but in this instance, *S. rubellum* outcompeted the *S. fuscum* to become the dominant *Sphagnum* species *c*. 10 yr post restoration (Poulin *et al*., 2013), with near complete *Sphagnum* coverage and net C sequestering in *c*. 15 yr (Nugent *et al*., 2018). It is important to note that the restored *Sphagnum* may not have the same pore structure as natural *Sphagnum* (McCarter & Price, 2013, 2015), which may not converge until decades after reestablishment if ever (Taylor & Price, 2015). As such, the evolution of *Sphagnum* and *Sphagnum*‐dominated peatland ecohydrology over time remains a gap in our knowledge.

As we look to improving our restoration techniques, it will be important to incorporate *Sphagna* with the greatest resilience to drought as *Sphagnum* re‐establishes. One successful restoration technique is moss layer transfer (Rochefort *et al*., 2006), and there is evidence to suggest that revegetation using mixed‐species *Sphagnum* plugs can yield a 99% establishment rate (Caporn *et al*., 2017). If employed in addition to rewetting, this can speed up the recovery rate of degraded peatlands (Allan *et al*., 2024) and those which have been subject to wildfire (Gage *et al*., 2024). Evidence is also building which shows that it is vital to restore the hummock‐hollow microtopography of a peatland in order to protect the restored landscape from ecohydrological extremes, such as drought (Loisel & Gallego‐Sala, 2022). While this will likely occur naturally over time, accelerating the microtopographical evolution of restored *Sphagnum* peatlands may increase the overall resilience of the peatland to the greater occurrence and severity of droughts in an era of climate change.

## V. Future work

*Sphagnum* is a globally important genus, distributed across all continents except Antarctica (Fig. 1). Despite this, research has focused on North America and Europe (Fig. 2), and although this undoubtedly encompasses core regions of *Sphagnum*'s range, large areas of the globe are not adequately represented. There is scope to study drought responses of *Sphagnum* from South America, Asia and Oceania in more detail. Representation also applies to the species which are explored in the literature, with species, such as *S. fallax* and *S. capillifolium* appearing in multiple studies, but other widely distributed species, such as *S. palustre* studied comparatively rarely. While some traits, such as the ecohydrological responses to drought, seem to be well conserved across hummock‐lawn ecotypes, other responses, such as photosynthesis, are less well delineated along those lines, which makes extrapolation form one species to another more difficult and highlights the importance of studying a broad spectrum of *Sphagnum* species.

Further research is needed on the recovery of *Sphagnum* from drought. Recovery is studied less often and for shorter periods than drought or desiccation, and this will become increasingly important as drought frequency rises. Likewise, repeated cycles of drought and recovery need to be understood in more detail. Some important work has been carried out in this area, but more is needed. This will enable us to identify thresholds and tipping points in these vital ecosystems with greater accuracy, which will improve peatland hydrological management.

There are important questions to address, regarding what evolutionary and physiological traits are key for enhancing recovery. At what scale are specific traits most influential? What matters more: plasticity of traits within populations, or variation between?

Our understanding of how *Sphagnum* physiology is linked to peatland hydrology is growing, but more work is needed. Research priorities include focusing on questions, such as How will drought under the future climate affect the physiology of *Sphagnum*? What are the feedbacks and buffers against drought provided by *Sphagnum* colonies in peatlands? When we restore *Sphagnum* peatlands, how can we exploit these buffers to restore in the most sustainable way, and how will this vary between geographic regions and *Sphagnum* species?

Timescales are also very important, and there is always a need for long‐term studies. Those that exist have provided essential insights into how peatland communities change with climate. Historic changes have been inferred through palaeoecological techniques, but with recent work suggesting that the bio‐climatic envelope of *Sphagnum* is under threat in many areas (Ritson *et al*., 2025), there are opportunities for experimental work to investigate how *Sphagnum* will respond to the boundaries of this envelope and whether long‐term exposure might facilitate adaptation in *Sphagnum* to enable its persistence.

How do we integrate all this information to think about how best to reintroduce *Sphagnum* for peatland restoration? The lessons learned regarding the damage to peatlands from extraction have led to a drive to cultivate *Sphagnum* sustainably, through paludiculture (see Pacheco‐Cancino *et al*., 2024). There is a similar opportunity to use the knowledge we have to ensure we create a *Sphagnum* layer that not only regenerates but also remains resilient to disturbance as the ecosystem develops. We must re‐establish *Sphagnum* communities that not only have species diversity but also trait diversity that aids in drought resistance and resilience (Robroek *et al*., 2024). Finally, we need to use experimental data to improve model representation of *Sphagnum* C cycling and hydrology in order to support decisions on land management and climate mitigation.

## Competing interests

None declared.

## Author contributions

BK, GDC, MGE, JPR and ELS contributed to the conceptualisation. BK, DMA, GDC, MGE, CDF, AJ, JL, CPRM, NO, JPR, BJMR, LR, ELS, YT, MRT and JMW contributed to the writing‐ original draft. BK and YT contributed to the visualisation. BK, GDC, CPRM, NO, JPR, BJMR, ELS and JMW contributed to the writing – review and editing. With the exception of the first author, all authors are listed alphabetically by last name.

## Disclaimer

The New Phytologist Foundation remains neutral with regard to jurisdictional claims in maps and in any institutional affiliations.
